# Persisting Smell and Taste Disorders in Patients Who Recovered from SARS-CoV-2 Virus Infection—Data from the Polish PoLoCOV-CVD Study

**DOI:** 10.3390/v14081763

**Published:** 2022-08-12

**Authors:** Michał Chudzik, Mateusz Babicki, Agnieszka Mastalerz-Migas, Joanna Kapusta

**Affiliations:** 1 Department of Internal Medicine and Geriatric Cardiology, Medical Centre for Postgraduate Education, 01-813 Warsaw, Poland; 2 Department of Family Medicine, Wroclaw Medical University, 51-141 Wrocław, Poland; 3 Department of Internal Medicine and Cardiac Rehabilitation, Medical University of Lodz, 70-445 Lodz, Poland

**Keywords:** COVID-19, long COVID, SARS-CoV-2, post-acute COVID-19 syndrome, smell and taste disorders, persistence, smell, taste

## Abstract

In the majority of cases, patients infected with the SARS-CoV-2 virus experience a complete resolution of symptoms within six weeks of acquiring the infection, but an increasing number of patients report persistent symptoms. This study aimed to analyse the prevalence of self-reported smell and/or taste disorders (STDs) in a group of convalescent patients after infection with the SARS-CoV-2 virus and to identify risk factors for the disease. The study included 2218 COVID-19 convalescents after both inpatient and outpatient treatment. The sample group was analysed with regard to chronic diseases, place of isolation and clinical symptoms occurring during COVID-19 along with their duration. The assessment also included the most common symptoms of COVID-19 and the severity of the disease course. A total of 98 patients reported persistent smell and taste disorders up to three months after the end of isolation (67.4% of men and 32.6% of women). The mean age of the participants was 53.8 ± 13.5 years (49.19 ± 14.68 in patients with an STD vs. 54.01 ± 13.44 in patients without an STD). The patients treated for COVID-19 at home (*p* < 0.001) constituted almost the entire group of patients with persistent smell and taste disorders (97%). Among the patients with persistent smell and taste disorders, 57.1% suffered from at least one chronic condition (vs. 71.4% of patients without an STD). In patients with an STD, the number of symptoms per patient was higher than in the other group at 8.87 ± 3.65 (*p* = 0.018), while the most common clinical symptoms during the acute phase of COVID-19 were smell and taste disorders (84%) (*p* < 0.001), significant weakness (70%), headache (60%), cough (55%), arthralgia (51%) (*p* = 0.034) and back muscle pain (51%). Based on the results obtained, the following conclusions were drawn: the risk of developing persistent smell and taste disorders after COVID-19 is greater in younger people with less comorbidities and a higher number of symptoms during the acute phase of COVID-19. The risk is associated with clinical symptoms occurring during the acute phase of COVID-19, i.e., smell and taste disorders and arthralgia. In addition, this risk is higher in patients receiving outpatient treatment for COVID-19.

## 1. Introduction

The clinical symptoms of COVID-19, a disease caused by the SARS-CoV-2 virus, most often resemble a mild cold and include rhinitis, cough and a sub-febrile state. Unfortunately, there are also cases of severe pneumonia with respiratory failure [1,2]. Smell and taste disorders (STDs) [3,4,5,6,7,8,9] are equally common and important symptoms of COVID-19. These include a decline in or loss of smell (hyposmia and anosmia) and/or taste (hypogeusia and ageusia). They are usually reported within the first three days of the first manifestation of the disease [6,10] in up to one-fourth of cases [11,12].

Before the COVID-19 pandemic, the prevalence of olfactory dysfunction in upper respiratory tract infections in the general population was approximately 3–20% [13,14]. The percentage of patients with COVID-19 experiencing smell and taste disorders is approximately 41% and 62%, respectively [12,15,16].

With the emergence of an increasing number of COVID-19 cases, the British Rhinological Society (BRS) and the British Association of Otorhinolaryngology—Head and Neck (ENTUK) were the first to observe and reveal that the loss of smell and/or taste could be an important symptom in disease diagnosis, pointing to SARS-CoV-2 infection. These initial findings were confirmed by subsequent observations [17,18,19,20].

Even though post-infectious smell disorders can be secondary to bacterial or fungal infection, they are most commonly caused by viruses [17,21,22,23]. The clinical course of olfactory impairment caused by COVID-19 is still unclear. Available evidence suggests that the most likely cause of anosmia during COVID-19 is the altered function of olfactory sensory neurons, their damage and, as a consequence, their death. Other mechanisms may also occur, including focal mucosal oedema and respiratory obstruction related to the inflammatory process in the olfactory epithelium [12,24]. Olfactory function impairment is usually accompanied by gustatory function impairment [14,25]. As in smell dysfunction, the pathogenesis of taste dysfunction in COVID-19 may involve indirect damage to taste receptors due to infection and the development of local inflammation [12]. This may be due to the report that the ACE2 receptor is highly expressed in epithelial cells, thus making the oral mucosa a possible site of entry for the SARS-CoV-2 virus [17,26]. According to NICE (National Institute for Health and Care Excellence), the condition of persistence of complaints and/or symptoms up to three months after infection—in the absence of an alternative diagnosis [27]—is referred to as long COVID syndrome or post-COVID-19 syndrome. According to the definition adopted by the WHO, this syndrome occurs in people after the disease and usually lasts up to 12 weeks. Its symptoms persist for at least two months and cannot be attributed to another cause [28]. In available observational studies [29,30] and cohorts, it was observed that it mainly affects outpatients [31,32,33].

In addition to fatigue and dyspnoea, smell and/or taste disorders are considered to be the main symptoms of the syndrome [34,35]. In many cases, these dysfunctions resolve within 7–14 days. However, in at least 10% of cases, patients continue to report persistent impairment after 4–6 weeks [17,22,36,37].

Long-term olfactory impairment can predispose patients to the development of medical conditions such as depression and cognitive impairment, which consequently worsens patients’ quality of life [14,24,38].

Therefore, this study aimed to analyse the prevalence of self-reported smell and/or taste disorders among a group of convalescent patients and to identify risk factors for the disease.

## 2. Materials and Methods

### 2.1. Methodology

The study included 2218 patients (808 women and 1410 men) with a history of COVID-19 (resolution of acute clinical symptoms—at least 14 days after last symptoms) who were part of the PoLoCOV-CVD (Polish Long-COVID Cardiovascular) study based on the analysis of data obtained from the STOP-COVID registry (study identifier: ClinicalTrials.gov—NCT05018052). The patient follow-up period spanned from 1 September 2020 to 30 September 2021 and included both patients who received hospital treatment and those who received outpatient treatment. The medical information in the registry refers to patients visiting healthcare centres because of persistent clinical symptoms after SARS-CoV-2 infection recovery. The sample group was analysed in relation to persistent smell and taste disorders.

This study used data obtained from the “0” appointment (first visit) and data obtained after 3 months. During the “0” appointment, information was collected from the patient regarding sociodemographics, chronic diseases, place of isolation and clinical symptoms occurring during COVID-19 along with their duration. Weight and height measurements were also taken to calculate the body mass index (BMI). In addition, patients completed health questionnaires and underwent a physical examination. Influenza and COVID-19 vaccination (after the introduction of COVID-19 vaccination in Poland, without regard to the type of vaccine used) was also evaluated, as well as the most common symptoms of COVID-19, i.e., fever/hypothermia, cough, shortness of breath, smell/taste/hearing dysfunction, weakness, chest/back/leg pain, headache/arthralgia, diarrhoea, vomiting, chills and blood pressure disorders (high blood pressure and/or dysregulation of well-controlled hypertension). The patients were also asked to provide a subjective rating of COVID-19 severity on a scale of 0 to 3 points. In the next step, the data obtained were used to classify the patients into separate groups according to the assessed disease severity given by them, where criteria included:

(A) 0 points (asymptomatic/mild course)
  - Asymptomatic course;
  - Parainfluenza symptoms lasting no more than 3 days.
(B) 1 point (mild course)
  - Home isolation;
  - Subjective patient rating of “1” on a scale of 0 to 3 points;
  - Duration of symptoms—less than 7 days.
(C) 2 points (moderate course)
  - Subjective patient rating of “2” or “3” on a scale of 0 to 3 points;
  - Duration of symptoms—7 to 14 days;
  - Presence of dyspnoea and fever ≥ 38 °C.
(D) 3 points (severe course)
  - Hospitalisation with a diagnosis of: pneumonia, respiratory failure, intensive care unit treatment, ventilator support, thromboembolic complications during hospitalisation/home isolation;
  - Or, in the case of home isolation: symptoms persisting for more than 14 days, temperature above 38 °C, dyspnoea, saturation < 94% persisting for at least 3 days, subjective patient rating of “3” on a scale of 0 to 3 points [39,40].

At the next follow-up appointment (3 months later), the patients completed a questionnaire that collected information on symptoms persisting up to 3 months after the end of isolation. Based on the rating given by the patients, smell and/or taste disorders were assessed after 3 months as disorders persisting continually from the onset of infection, higher than 50% compared to the pre-COVID-19 period.

The inclusion criteria were:

(a) Diagnosis of SARS-CoV-2 virus infection (in accordance with current guidelines of the Ministry of Health of the Republic of Poland);
(b) Age ≥ 18 years;
(c) Absence of contraindications to participating in the study;
(d) Full recovery (resolution of acute clinical symptoms—at least 2 weeks after last symptoms);
(e) Written consent to participate in the study.

Prior to participation, the patients were informed of the study objectives and how the study would be conducted, after which they provided informed consent to participate in the study. The study was conducted according to the guidelines of the Declaration of Helsinki and approved by the Bioethics Committee of Wroclaw Medical University, Poland (approval number 232/2022).

### 2.2. Statistical Analysis

Basic descriptive statistics were used to describe the sample group. The analysis pertained to qualitative, quantitative and ordinal variables. The groups of patients with and without smell and taste disorders were compared using the chi-square test for qualitative variables and the Mann–Whitney U test for quantitative and ordinal variables. The propensity-score-matched analysis, in which matching baseline variables included age and sex, was performed to minimise the baseline differences between patient groups. Matching baseline variables was performed at a ratio of 1:10.

Logistic regression analysis was used to assess risk factors for the development of persistent smell and taste disorders. Univariate models were constructed, where the dependent (explanatory) variable was the presence of smell and taste disorders and the explanatory variables were sociodemographic variables (age, gender), influenza and COVID-19 vaccination status, chronic conditions, including their number, and the course of COVID-19 (place of isolation, symptoms during the disease, their number and duration and disease severity).

Multivariate logistic regression models with best subset selection were subsequently built for the explanatory variables using the Akaike criterion. In these models, statistically significant (*p* < 0.05) variables obtained through univariate analysis were the explanatory variables. Results are presented as OR (odds ratio) with 95% Cl (confidence interval) and *p*-value, where *p* < 0.05 was assumed. The analysis was conducted with the use of Statistica 13.0 by StatSoft.

## 3. Results

### Characteristics of the Study Group

A total of 2218 patients (36.4% women and 63.6% men) were included in the analysis, of whom 98 reported persistent smell and taste disorders (67.4% men and 32.6% women). The mean age of the participants was 53.8 ± 13.5 years (*p* < 0.001). Based on the BMI, 692 (31.2%) patients were diagnosed with obesity, and obesity was also diagnosed in 22.5% (*p* = 0.042) of the group with persistent smell and taste disorders. Among the patients with persistent smell and taste disorders, 57.1% of patients had at least one chronic disease (*p* = 0.002) (with an average of 1.29 ± 1.44 conditions per patient (*p* = 0.011)), with the most common diseases in this group being hypertension (24.5%) (vs. 37.8% in pts without an STD; *p* = 0.007), thyroid diseases (17.4%), hyperlipidaemia (14.3%) and diabetes (11.2%). In addition, in 2021, 7.5% of patients in the total group were vaccinated against influenza and 22.3% against COVID-19 (3.1% and 73.9 in patients with an STD). A detailed description of the sample group, broken down into patients with/without persistent smell and taste disorders, is presented in Table 1.

The patients treated for COVID-19 at home (*p* < 0.001) constituted almost the entire group of patients with persistent smell and taste disorders (97%). The analysis of the disease course shows that the occurrence of persistent smell and taste disorders was most commonly reported after the second wave of the pandemic, i.e., 67 (68.4%) patients (*p* = 0.003). After the next waves of the pandemic, the number of patients reporting persistent smell and taste disorders decreased (wave 3–21.4 per cent, wave 4–10.2 per cent of patients). There was no difference in the incidence of smell and taste disorders between mild, moderate and severe courses of COVID-19. In addition, in the group of patients with a persistent STD, the number of symptoms per patient was higher than in the other group at 8.87 ± 3.65 (*p* = 0.018), while the most common clinical symptoms during the acute phase of COVID-19 were smell and taste disorders (84%) (*p* < 0.001), significant weakness (70%), headache (60%), cough (55%), arthralgia (51%) (*p* = 0.034) and back muscle pain (51%), leg muscle pain (49%), fever > 37.5 °C, dyspnoea (46%) and chest pain (46%). The duration of reported symptoms was approximately 10 days (10.43 ± 5.49). A detailed summary of the data is shown in Table 2. The detailed distribution of the groups after propensity score matching is shown in Tables S1 and S2, which were added as Supplementary Materials.

The univariate analysis showed that the risk of developing persistent smell and taste disorders after COVID-19 was associated with the obesity (*p* = 0.04), age (*p* = 0.001), number of comorbidities (*p* = 0.01) and number of symptoms during COVID-19 (*p* = 0.01). The risk was greater in younger people with less comorbidities and a higher number of symptoms during the acute phase of COVID-19. The analysis of individual clinical symptoms showed that smell and taste disorders (*p* = 0.000) and arthralgia (*p* = 0.03) occurring during the acute phase of COVID-19 were associated with a higher risk of developing a persistent STD. It was also observed that people with an STD suffered from hypertension less frequently (*p* = 0.009). Furthermore, the risk of developing an STD was shown to be significantly higher in patients treated for COVID-19 at home (*p* = 0.002).

**Figure 1:**
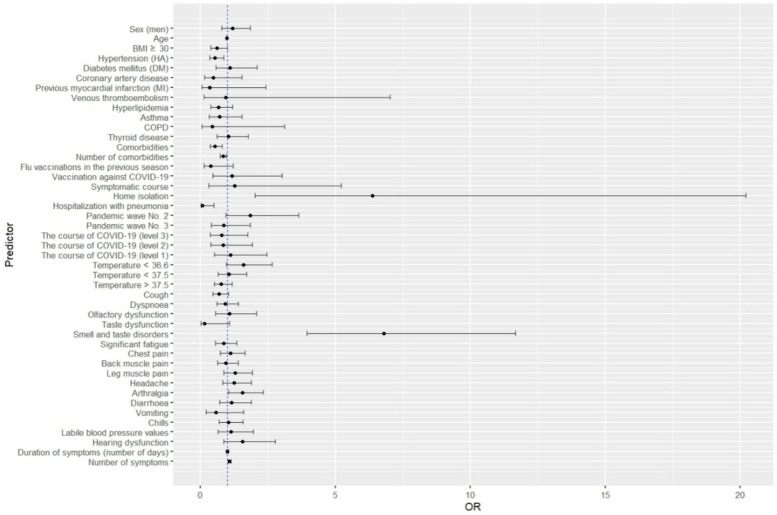
Graphical representation of univariate analysis determining the effect of sociodemographic variables, chronic diseases, influenza and COVID-19 vaccination and COVID-19 course on the risk of developing persistent smell and taste disorders.

The propensity-score-matched analysis, in which matching baseline variables included age and sex, confirmed a statistically significant relationship between home isolation (*p* = 0.043), smell and taste disorders (*p* < 0.001), arthralgia (*p* = 0.016) and the number of symptoms (*p* = 0.02) occurring in the acute phase of COVID-19 with a higher risk of developing a persistent STD. In addition, it showed an increased risk of developing persistent smell and taste disorders after COVID-19 in patients with leg muscle pain (*p* = 0.04) and a decreased risk in patients contracting the virus in pandemic wave 3 (*p* = 0.016). Table 3 and Figure 1 show a detailed summary of the data.

**Figure 2:**
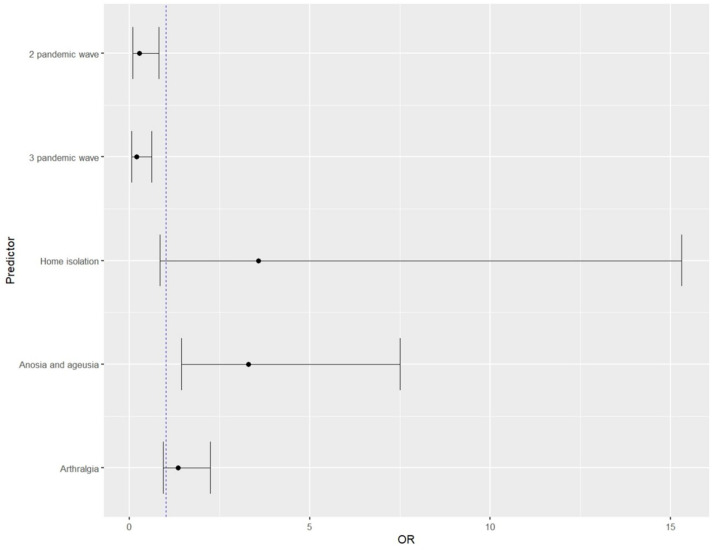
Graphical representation of multivariate analysis based on a model incorporating statistically significant predictors from univariate analysis determining the risk of developing persistent smell and taste disorders.

In multivariate analysis, the risk of developing persistent smell and taste disorders after COVID-19 was shown to be associated with patients treated for COVID-19 at home. Among the clinical symptoms occurring during the acute phase of COVID-19, smell and taste disorders and arthralgia were associated with a higher risk of developing a persistent STD. The propensity-score-matched analysis, in which matching baseline variables included age and sex, confirmed a statistically significant relationship between home isolation (*p* = 0.043), smell and taste disorders (the strongest independent risk factor for an STD—over 3.5 times; *p* = 0.004) and arthralgia (*p* = 0.05) occurring in the acute phase of COVID-19 with a higher risk of developing a persistent STD. In addition, it showed a decreased risk of developing persistent smell and taste disorders after COVID-19 in patients from wave 2 and 3 of the pandemic. A detailed summary of the data is shown in Table 4 and Figure 2.

## 4. Discussion

A significant proportion of SARS-CoV-2 infections may be mild or asymptomatic, with a sudden loss of sense of smell or taste being reported as an isolated symptom in 3% of cases [5,41,42,43]. In this study, in the acute phase of COVID-19, 45% of the patients were observed to have smell and taste disorders, including 83.67% of the patients in the group with a persistent STD. In most cases, patients experience a complete resolution of symptoms after 2 to 6 weeks [33], but an increasing number of people are reporting symptoms persisting for even more than 3 months after recovering from COVID-19. This study analysed the occurrence of smell and taste disorders in patients with long COVID. According to World Health Organization (WHO) recommendations, patients with a positive test result for SARS-CoV-2, without risk factors and with a mild course of COVID-19, can undergo treatment at home during isolation [43,44]. In the available literature, it was observed that these long-term symptoms are very common among outpatients [30,31,32,33]. Similar observations were noted by Logue JK and Klein H [45,46], who analysed patients with mild-to-moderate COVID-19 treated on an outpatient basis [33]. In this study, 2218 patients (36.4% women and 63.6% men) were included in the analysis, of whom 4.42% reported persistent smell and taste disorders. Patients with a mild course of COVID-19 who were treated at home constituted almost the entire group of patients with persistent smell and taste disorders (97%). A study by Athanasia Printza et al. reported no difference in the prevalence of smell and taste disorders between mild, moderate and severe courses of the COVID-19 disease. This notwithstanding, previous studies pointed to a higher prevalence of chemosensory deficits in outpatients compared to inpatients [47,48]. One of the reasons could be that those being hospitalised have more severe complications with life-threatening conditions, and less attention has been given to STDs. The analysis of recovery time for patients who regained their sense of smell showed that patients with hyposmia recovered faster compared to patients with anosmia. This is consistent with the data presented by Lechien et al., who found that less severe olfactory loss was significantly associated with earlier recovery [49,50]. In the study presented in this paper, the mean duration of reported symptoms was approximately 10 days (10.72 ± 5.74), but the patients were not differentiated by STD severity.

Lara Bull-Otterson et al. observed that post-COVID-19 smell and taste disorders were more common in patients under 65 years of age [51]. In this study, the mean age of subjects with an STD was 49.19 ± 14.68 years, confirming these observations. These patients were young and had less comorbidities than other people more severely clinically affected by COVID-19. The results we obtained can be explained, for example, on the basis of the immune response. People with a good and efficient immune response demonstrate prolonged immune-mediated disease of smell cells, prolonging symptoms [52]. Moreover, it is presumed that younger patients, who required hospitalisation less frequently, received mainly symptomatic treatment of the cold (without corticosteroids) or, in the case of asymptomatic patients in quarantine, no medication was used [53]. The available literature contains studies demonstrating the benefits of short-term topical or oral use of corticosteroids [5,24,53,54,55] and olfactory training [56] involving repeated exposure to smells [55,57]. The quality of evidence demonstrating the effectiveness of these therapies, due to the size of the study populations, varies significantly [17], and to date, there have been no large-scale studies assessing their effectiveness [5,24,53,54,55]. Furthermore, oral corticosteroids were initially contraindicated by the World Health Organization as a form of treatment in this group of patients. The approach to steroid treatment began to change as more evidence emerged pointing to a significant reduction in mortality in patients with severe COVID-19 [24,58,59]. Furthermore, a study by Hyun Kim et al. shows that topical corticosteroid administration 2 and 4 weeks after treatment can accelerate recovery from olfactory dysfunction caused by COVID-19 infection compared with non-administration of corticosteroids (*p* < 0.0001). The results support the idea that the olfactory dysfunction observed in COVID-19 is mainly caused by an inflammatory process in the olfactory epithelium, and intranasally administered corticosteroids may have a beneficial anti-inflammatory effect [60]. Furthermore, it is believed that, in addition to reducing local inflammation, intranasal corticosteroids may improve the sense of smell by impacting on Na-K-ATPase enzyme activity and modulating the neuronal function of the olfactory receptor [14,61]. This means that although additional research is required to determine treatment outcomes among COVID-19 patients with post-infectious smell and taste loss, it is reasonable to build on previous treatment guidelines and recommend topical corticosteroids and olfactory training as first-line therapies for these patients [53] in the early acute phase of COVID-19 [24]. Nguyen NN et al. [62] observed that 30 out of 125 (24.0%) patients who reported smell and/or taste disorders during the initial acute phase of COVID-19 reported a persistence of these symptoms 6 months after onset. Similar observations in their study were made by Tan BKJ et al. [63]. Additionally, in the study presented in this paper, the risk of developing persistent smell and taste disorders after COVID-19 was higher in patients with clinical symptoms during the acute phase of COVID-19, i.e., smell and taste disorders and arthralgia. Most studies have not found any association of comorbidities with the persistence of olfactory dysfunction [49], confirming the results of the analysis presented in this study, but a recent study by Amer MA et al. revealed that comorbidities are associated with poorer olfactory recovery in patients with allergic rhinitis, smokers and patients with hypertension [50,64].

The study by Karaarslan F. et al. [65] showed the effect of obesity on the persistence of symptoms associated with SARS-CoV-2 infection. Khan A.S. et al. [66] investigated the relationship between obesity and COVID-19. They observed that obesity may worsen the identification of olfactory and taste disorders caused by SARS-CoV-2 virus infection. Patel et al. [67] also found that a high BMI was associated with subjective olfactory dysfunction in obese patients. In our study, 77.6% of patients with persistent olfactory and taste disorders had a BMI < 30.

Moreover, the analysis of the disease course shows a decreased risk of developing persistent smell and taste disorders after COVID-19 in patients from wave 2 to 3 of the pandemic. As the pandemic progressed, it was shown that the percentage of people experiencing this dysfunction actually decreased, which confirms the observations of Boscolo-Rizzo P. et al. [68] showing the prevalence and the severity of smell and taste disorders after COVID-19 has dropped significantly with the advent of the Omicron variant. However, our dataset did not include patients suffering during the fifth wave of the pandemic; therefore, the results may differ.

The increasing number of individuals who have recovered from COVID-19 with persistent smell and/or taste disorders makes it necessary to identify predisposing factors for the observed disturbances to provide a better explanation of the mechanism of action of the SARS-CoV-2 virus. Therefore, this study aimed to analyse the prevalence of self-reported smell and/or taste disorders among a group of convalescent patients and to identify risk factors for the disease.

Strengths and limitations of the study. The presented study was limited to interventions provided in primary care. It focused on patients who presented to a medical facility on their own, meaning that not all patients who had suffered from COVID-19 were included. The methodology used in the study is also a limitation because chemosensory dysfunction was not documented with olfactory or taste tests but was based on an interview with the patient. Another limiting factor is the relatively short follow-up period and the small sample size of the subgroups of patients with chemosensory loss. Differences in the two investigated subgroups may be detectable in larger groups of participants. It should be emphasised that in the study presented in this paper, the female population was almost half the size of the male population, so the results should not be translated to the entire population of COVID-19 convalescents. Furthermore, the authors of this study did not consider the impact of taking chronic medications or medications during and after infection, which may affect the occurrence and persistence of smell and taste disorders in long COVID, as well as the syndrome itself.

The strength of the presented study is that it compiles data from one of the largest registries of patients who suffered from SARS-CoV-2 virus infection (STOP-COVID), where most patients are those who received outpatient treatment for COVID-19. The sample group makes it possible to assess the impact of the place of isolation on the risk of developing the long-COVID syndrome and clinical symptoms during the course of the disease. This is very important because most COVID-19 patients without risk factors and with a mild course of the disease are treated at home. In addition, in the group with persistent smell and/or taste disorders, the patients were given recommendations for rehabilitation therapy based on taste and smell training. The STOP-COVID programme is still in progress, and a follow-up is planned in the future to evaluate the effectiveness of the methods used.

## 5. Conclusions

Based on the results obtained, the following conclusions were drawn: the risk of developing persistent smell and taste disorders after COVID-19 is greater in younger people with less comorbidities and a higher number of symptoms during the acute phase of COVID-19. The risk is associated with clinical symptoms occurring during the acute phase of COVID-19, i.e., smell and taste disorders and arthralgia. Moreover, this risk is higher in patients receiving outpatient treatment for COVID-19, which may be related to the provision of different treatments to these patients than patients admitted to hospital wards with a severe disease course where, among other methods, steroid treatment is used. The increasing number of COVID-19 convalescent patients with persistent smell and/or taste disorders combined with the absence of an effective method for the treatment and rehabilitation of these patients necessitate the identification of predisposing factors for the observed dysfunctions, which will make it possible to better explain the mechanism of action of the SARS-CoV-2 virus and, therefore, introduce effective adjunctive therapies.

## Figures and Tables

**Table 1:** Patient characteristics—differences between groups with and without persistent smell and taste disorders.

	Smell and Taste Disorders (*n* = 98)	No Smell and Taste Disorders (*n* = 2120)	*p*-Value *	Total (*n* = 2218)
Age **	49.19 ± 14.68	54.01 ± 13.44	<0.001	53.80 ± 13.53
**Women**	32 (32.65%)	776 (36.60%)	0.426	808 (36.43%)
**Men**	66 (67.35%)	1344 (63.40%)		1410 (63.57%)
Weight **	76.58 ± 15.64	79.75 ± 17.30	0.091	79.61 ± 17.24
Height **	169.13 ± 8.38	169.11 ± 9.16	0.953	169.11 ± 9.13
**BMI < 30**	76 (77.55%)	1409 (67.77%)	0.042	1485 (66.95%)
**BMI ≥ 30**	22 (22.45%)	670 (32.23%)		692 (31.20%)
Number of comorbidities **	1.29 ± 1.44	1.70 ± 1.62	0.011	1.68 ± 1.62
**Comorbidities**	56 (57.14%)	1513 (71.37%)	0.002	1569 (70.74%)
**Hypertension (HT)**	24 (24.49%)	802 (37.83%)	0.007	826 (37.24%)
**Thyroid disease**	17 (17.35%)	357 (16.84%)	0.675	374 (16.86%)
**Hyperlipidaemia**	14 (14.29%)	426 (20.09%)	0.158	440 (19.84%)
**Diabetes mellitus (DM)**	11 (11.22%)	219 (10.33%)	0.773	230 (10.37%)
**Coronary artery disease**	3 (3.06%)	131 (6.18%)	0.205	134 (6.04%)
**Previous myocardial infarction (MI)**	1 (1.02%)	64 (3.02%)	0.001	65 (2.93%)
**Heart failure**	0 (0.00%)	26 (1.23%)	0.270	26 (1.17%)
**Venous thromboembolism**	1 (1.02%)	23 (1.08%)	0.951	24 (1.08%)
**Asthma**	7 (7.14%)	210 (9.91%)	0.368	217 (9.78%)
**COPD**	1 (1.02%)	50 (2.36%)	0.387	51 (2.30%)
**Flu vaccinations in the previous season**	3 (3.06%)	163 (7.69%)	0.087	166 (7.48%)
**Vaccination against COVID-19 (*n* = 697)/** **(*n* = 23 for smell and taste disorders)**	17 (73.91%)	477 (70.77%)	0.744	494 (22.27%)

BMI—body mass index, HT—hypertension, DM—diabetes mellitus, MI—myocardial infarction, COPD—chronic obstructive pulmonary disease, * chi-square test—distribution among patients with/without persistent smell and taste disorders, ** Mann–Whitney U test.

**Table 2:** COVID-19 course analysis in groups with and without persistent smell and taste disorders.

	Smell and Taste Disorders (*n* = 98)	No Smell and Taste Disorders (*n* = 2120)	*p*-Value *	Total (*n* = 2218)
**Pandemic wave**				
**2**	67 (68.37%)	1087 (51.27%)	0.003	1154 (52.03%)
**3**	21 (21.43%)	733 (34.58%)		754 (33.99%)
**4**	10 (10.20%)	300 (14.15%)		310 (13.98%)
**Course of COVID-19**				
**0**	8 (8.16%)	157 (7.48%)	0.542	165 (7.44%)
**1**	35 (35.71%)	616 (29.33%)		651 (29.35%)
**2**	25 (25.51%)	580 (27.62%)		605 (27.28%)
**3**	30 (30.61%)	747 (35.57%)		777 (35.03%)
**Home isolation**	95 (96.94%)	1765 (83.25%)	<0.001	1860 (83.86%)
**Hospitalisation without pneumonia**	0 (0.00%)	33 (1.56%)	0.213	33 (1.49%)
**Hospitalisation with pneumonia**	1 (1.02%)	278 (13.11%)	0.004	279 (12.58%)
**Hospitalisation with ICU**	0 (0.00%)	19 (0.90%)	0.346	19 (0.86%)
**Symptoms during COVID-19**				
**Temperature < 36.6**	20 (20.41%)	294 (13.87%)	0.056	314 (14.16%)
**Temperature < 37.5**	23 (23.47%)	477 (22.50%)	0.822	500 (22.54%)
**Temperature > 37.5**	46 (46.94%)	1132 (53.40%)	0.210	1178 (53.11%)
**Cough**	54 (55.10%)	1360 (64.15%)	0.068	1414 (63.75%)
**Dyspnoea**	45 (45.92%)	1014 (47.83%)	0.711	1059 (47.75%)
**Olfactory dysfunction**	10 (10.20%)	205 (9.67%)	0.816	215 (9.69%)
**Taste dysfunction**	1 (1.02%)	139 (6.56%)	0.027	140 (6.31%)
**Smell and taste disorders**	82 (83.67%)	912 (43.02%)	<0.001	994 (44.82%)
**Significant fatigue**	69 (70.41%)	1555 (73.35%)	0.520	1624 (73.22%)
**Chest pain**	45 (45.92%)	923 (43.54%)	0.642	968 (43.64%)
**Back muscle pain**	50 (51.02%)	1115 (52.59%)	0.760	1165 (52.52%)
**Leg muscle pain**	48 (48.98%)	907 (42.78%)	0.225	955 (43.06%)
**Headache**	59 (60.20%)	1166 (55.00%)	0.311	1225 (55.23%)
**Arthralgia**	50 (51.02%)	851 (40.14%)	0.034	901 (40.62%)
**Diarrhoea**	21 (21.43%)	409 (19.29%)	0.601	430 (19.39%)
**Vomiting**	4 (4.08%)	146 (6.89%)	0.279	150 (6.76%)
**Chills**	36 (36.73%)	761 (35.90%)	0.865	797 (35.93%)
**Labile blood pressure values**	16 (16.33%)	311 (14.67%)	0.651	327 (14.74%)
**Hearing dysfunction**	14 (14.29%)	206 (9.72%)	0.139	220 (9.92%)
Duration of symptoms (number of days) **	10.43 ± 5.49	10.73 ± 5.75	0.578	10.72 ± 5.74
Number of symptoms **	8.87 ± 3.65	7.91 ± 3.61	0.018	7.95 ± 3.62

ICU—intensive care unit, * chi-square test—distribution among people with/without persistent smell and taste disorders. ** the Mann–Whitney U test.

**Table 3:** Univariate analysis determining the effect of sociodemographic variables, chronic diseases, influenza and COVID-19 vaccination and COVID-19 course on the risk of developing persistent smell and taste disorders.

The Whole Group						Propensity Score Matching			
		OR	95% CI for OR		*p*-Value	OR	95% CI for OR		
			Lower Limit	Upper Limit			Lower Limit	Upper Limit	*p*-Value
Sex (men)		1.191	0.774	1.833	0.427	0.913	0.521	1.600	0.751
Age		0.973	0.959	0.987	0.001	0.998	0.979	1.017	0.857
BMI ≥ 30		0.609	0.375	0.987	0.044	0.659	0.356	1.220	0.185
Hypertension (HT)		0.533	0.334	0.852	0.009	0.765	0.419	1.397	0.384
Diabetes mellitus (DM)		1.098	0.577	2.087	0.777	1.367	0.559	3.338	0.493
Coronary artery disease		0.479	0.150	1.534	0.215	0.407	0.054	3.063	0.383
Previous myocardial infarction (MI)		0.331	0.045	2.412	0.275	0.000	-	-	-
Heart failure		0.000	-	-	-	0.000	-	-	-
Venous thromboembolism		0.940	0.126	7.032	0.952	1.112	0.139	8.932	0.919
Hyperlipidaemia		0.663	0.373	1.179	0.161	0.864	0.427	1.746	0.683
Asthma		0.700	0.320	1.529	0.371	0.835	0.322	2.160	0.709
COPD		0.427	0.058	3.122	0.402	0.831	0.106	6.493	0.859
Thyroid disease		1.036	0.607	1.770	0.896				
Comorbidities		0.535	0.355	0.807	0.003	0.625	0.374	1.045	0.073
Number of comorbidities		0.833	0.720	0.963	0.014	0.837	0.696	1.007	0.059
Flu vaccinations in the previous season		0.379	0.119	1.210	0.101	0.221	0.029	1.628	0.138
Vaccination against COVID-19		1.171	0.456	3.011	0.744	1.133	0.654	2.873	0.654
Symptomatic course		1.255	0.301	5.222	0.755	2.031	0.268	15.389	0.492
Home isolation		6.370	2.007	20.215	0.002	3.441	0.832	14.392	0.043
Hospitalisation without pneumonia		0.000	-	-	-	0.000	-	-	-
Hospitalisation with pneumonia		0.068	0.009	0.492	0.008	0.195	0.026	1..443	0.109
Hospitalisation with ICU		0.000	-	-	-	0.000	-	-	-
Pandemic wave	2	1.851	0.939	3.637	0.075	0.562	0.258	1.223	0.146
	3	0.859	0.399	1.846	0.698	0.323	0.128	0.811	0.016
Course of COVID-19	3	0.788	0.355	1.752	0.361	0.682	0.272	1.708	0.546
	2	0.846	0.374	1.912	0.622	0.690	0.271	1.753	0.595
	1	1.115	0.507	2.452	0.290	0.785	0.318	1.938	0.971
Temperature < 36.6		1.593	0.960	2.643	0.072	1.478	0.775	2.821	0.236
Temperature < 37.5		1.056	0.655	1.704	0.822	1.088	0.601	1.969	0.779
Temperature > 37.5		0.772	0.515	1.159	0.212	0.782	0.469	1.302	0.344
Cough		0.686	0.456	1.031	0.070	0.794	0.474	1.330	
Dyspnoea		0.926	0.617	1.390	0.711	0.982	0.587	1.639	0.943
Olfactory dysfunction		1.062	0.543	2.074	0.861	1.067	0.468	2.440	0.875
Taste dysfunction		0.147	0.020	1.062	0.057	0.000	-	-	-
Smell and taste disorders		6.788	3.946	11.678	0.000	5.885	2.948	11.746	<0.001
Significant fatigue		0.865	0.554	1.348	0.521	0.791	0.449	1.388	0.413
Chest pain		1.101	0.733	1.653	0.642	1.203	0.722	2.003	0.477
Back muscle pain		0.939	0.626	1.408	0.760	1.139	0.681	1.906	0.618
Leg muscle pain		1.284	0.856	1.925	0.227	1.713	1.026	2.864	0.039
Headache		1.238	0.819	1.872	0.312	1.267	0.736	2.183	0.392
Arthralgia		1.553	1.036	2.330	0.033	1.884	1.125	3.151	0.016
Diarrhoea		1.141	0.696	1.871	0.601	1.128	0.691	2.341	0.438
Vomiting		0.575	0.209	1.587	0.286	0.822	0.288	2.358	0.716
Chills		1.037	0.681	1.578	0.866	1.059	0.629	1.783	0.827
Labile blood pressure values		1.135	0.655	1.965	0.651	1.146	0.563	2.331	0.706
Hearing dysfunction		1.549	0.864	2.776	0.142	1.694	0.822	3.487	0.153
Duration of symptoms (number of days)		0.990	0.947	1.036	0.676	0.993	0.948	1.041	0.768
Number of symptoms		1.076	1.017	1.138	0.011	1.087	1.012	1.167	0.021

OR—odds ratio; CI—confidence interval; BMI—body mass index, HT—hypertension, DM—diabetes mellitus, MI—myocardial infarction, COPD—chronic obstructive pulmonary disease, ICU—intensive care unit.

**Table 4:** Multivariate analysis based on a model incorporating statistically significant predictors from univariate analysis on the risk of developing persistent smell and taste disorders.

	The Whole Group				Propensity Score Matching		
	OR	95% CI for OR		*p*-Value	OR	95% CI for OR	
		Lower Limit	Upper Limit			Lower Limit	Upper Limit
Comorbidities	0.590	0.384	0.906	**0.016**	---	---	---
Home isolation	4.085	1.271	13.129	**0.018**	3.566	0.830	15.312
Smell and taste disorders	3.454	1.856	6.429	**0.000**	3.285	1.439	7.493
Arthralgia	1.519	0.996	2.318	**0.052**	1.341	0.930	2.231
Pandemic wave 2	---	---	---	---	0.274	0.092	0.804
Pandemic wave 3	---	---	---	---	0.187	0.057	0.608

OR—odds ratio; CI—confidence interval.

## Data Availability

The data underlying this article cannot be shared publicly in order to maintain the privacy of individuals that participated in the study.

## Supplementary Materials

The following supporting information can be downloaded at: https://www.mdpi.com/article/10.3390/v14081763/s1. Table S1. Patient characteristics after propensity score matching—differences between the group with and without persistent smell and taste disorders. Table S2. COVID-19 course analysis in groups with and without persistent smell and taste disorders. Comparison after propensity score matching.
