# Supplementary material for: Persisting Smell and Taste Disorders in Patients Who Recovered from SARS-CoV-2 Virus Infection—Data from the Polish PoLoCOV-CVD Study
Source: Viruses. 2022 Aug 12;14(8):1763. doi: 10.3390/v14081763 (PMC9416276; doi:10.3390/v14081763)
Supplement: Supplementary file 1 [file viruses-14-01763-s001.zip › viruses-1842975-supplementary.pdf]

Table S1. Patient characteristics after propensity score matching – differences between the group with and without persistent smell and taste disorders.

	Smell and taste disorders (n = 98)	No smell and taste disorders (n=980)	p-value*
**Age****	49.19±14.68	28.5±13.02	0.698
**Women**	32 (32.65%)	353 (36.4)	0.863
**Men**	66 (67.35%)	637 (63.4)	
**Weight** **	76.58±15.64	78.19±18.04	0.372
**Height** **	169.13±8.38	168.87±8.63	0.401
**BMI <30**	76 (77.55%)	696 (70.6)	0.234
**BMI ≥30**	22 (22.45%)	284 (29.4)	
**Number of comorbidities** **	1.29±1.44	1.52±1.60	0.072
**Comorbidities**	56 (57.14%)	624 (63.7)	0.094
**Hypertension (HT)**	24 (24.49%)	276 (28.2)	0.467
**Thyroid disease**	17 (17.35%)	181 (18.5)	0.451
**Hyperlipidemia**	14 (14.29%)	170 (17.4)	0.814
**Diabetes mellitus (DM)**	11 (11.22%)	68 (6.9)	0.662
**Coronary artery disease**	3 (3.06%)	36 (3.7)	0.581
**Previous myocardial infarction (MI)**	1 (1.02%)	12 (1.2)	0.778
**Heart failure**	0 (0.00%)	13 (1.3)	0.710
**Venous thromboembolism**	1 (1.02%)	13 (1.3)	0.650
**Asthma**	7 (7.14%)	89 (9.08)	0.884
**COPD**	1 (1.02%)	18 (1.9)	0.756
**Flu vaccinations in the previous season**	3 (3.06%)	64 (6.6)	0.175
**Vaccination against COVID-19 (n=532) / (n=23 for smell and taste disorders)**	17 (73.91%)	369 (69.5)	0.652

BMI – Body Mass Index, HT – hypertension, DM – diabetes mellitus, MI – myocardial infarction, COPD – chronic obstructive pulmonary disease, * Chi-square test – distribution among patients with/without persistent smell and taste disorders, ** Mann–Whitney U test.

Table S2. COVID-19 course analysis in groups with and without persistent smell and taste disorders. Comparison after propensity score matching.

	Smell and taste disorders (n = 98)	No smell and taste disorders (n=980)	p-value*
Pandemic wave			
**2**	67 (68.37%)	617 (62.9%)	0.051
**3**	21 (21.43%)	293 (29.9%)	
**4**	10 (10.20%)	70 (7.2%)	
**Course of COVID-19**			
**0**	8 (8.16%)	78 (8.0)	0.853
**1**	35 (35.71%)	314 (32.0)	
**2**	25 (25.51%)	276 (28.2)	
**3**	30 (30.61%)	312 (31.8)	
**Home isolation**	95 (96.94%)	884 (90.2%)	0.115

**Hospitalisation without pneumonia**	0 (0.00%)	12 (1.2%)	0.778
**Hospitalisation with pneumonia**	1 (1.02%)	73 (7.4%)	0.128
**Hospitalisation with ICU**	0 (0.00%)	4 (0.5)	0.647
**Symptoms during COVID-19**			
**Temperature <36.6**	20 (20.41%)	142 (14.5%)	0.312
**Temperature <37.5**	23 (23.47%)	226 (23.1%$)	0.899
**Temperature > 37.5**	46 (46.94%)	527 (53.8%)	0.413
**Cough**	54 (55.10%)	612 (62.5)	0.457
**Dyspnoea**	45 (45.92%)	442 (45.1%)	0.952
**Olfactory dysfunction**	10 (10.20%)	99 (10.2%)	0.953
**Taste dysfunction**	1 (1.02%)	55 (5.7%)	0.092
**Smell and taste disorders**	82 (83.67%)	473 (48.3%)	<0.001
**Significant fatigue**	69 (70.41%)	738 (75.4%)	0.503
**Chest pain**	45 (45.92%)	452 (46.2%)	0.516
**Back muscle pain**	50 (51.02%)	526 (53.7%)	0.713
**Leg muscle pain**	48 (48.98%)	411 (42.0%)	0.051
**Headache**	59 (60.20%)	611 (62.3%)	0.470
**Arthralgia**	50 (51.02%)	404 (41.2%)	0.021
**Diarrhoea**	21 (21.43%)	187 (19.1%)	0.540
**Vomiting**	4 (4.08%)	72 (7.4%)	0.909
**Chills**	36 (36.73%)	378 (38.6%)	0.932
**Labile blood pressure values**	16 (16.33%)	134 (13.7%)	0.850
**Hearing dysfunction**	14 (14.29%)	95 (9.7%)	0.219
**Duration of symptoms (number of days)****	10.43±5.49	10.64±5.64	0.642
**Number of symptoms****	8.87±3.65	8.29±3.63	0.021

ICU – intensive care unit, *Chi-square test – distribution among people with/without persistent smell and taste disorders. ** the Mann–Whitney U test.
